# Supplementary material for: Wide-temperature-range thermoelectric n-type Mg3(Sb,Bi)2 with high average and peak zT values
Source: Nat Commun. 2023 Nov 16;14:7428. doi: 10.1038/s41467-023-43228-9 (PMC10654674; doi:10.1038/s41467-023-43228-9)
Supplement: Supplementary file 1 — Supplementary Information [file 41467_2023_43228_MOESM1_ESM.pdf]

Supplementary Information

Wide-temperature-range thermoelectric n-type $Mg_3(Sb,Bi)_2$ with high average and peak zT values

Jing-Wei Li,[1] Zhijia Han,[2] Jincheng Yu,[1] Hua-Lu Zhuang,[1,*] Haihua Hu,[1] Bin Su,[1] Hezhang Li,[1] Yilin Jiang,[1] Lu Chen,[1] Weishu Liu,[2] Qiang Zheng,[3] and Jing-Feng Li[1,*]

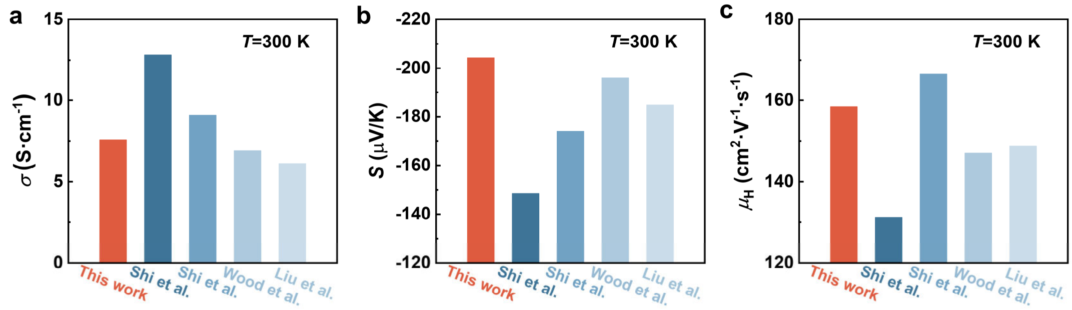

Supplementary Figure 1. Comparison of electrical transport properties. The comparison of (a) electrical conductivity, (b) Seebeck coefficient, and (c) Hall mobility in this work with previously reported results[15,21,27,28].

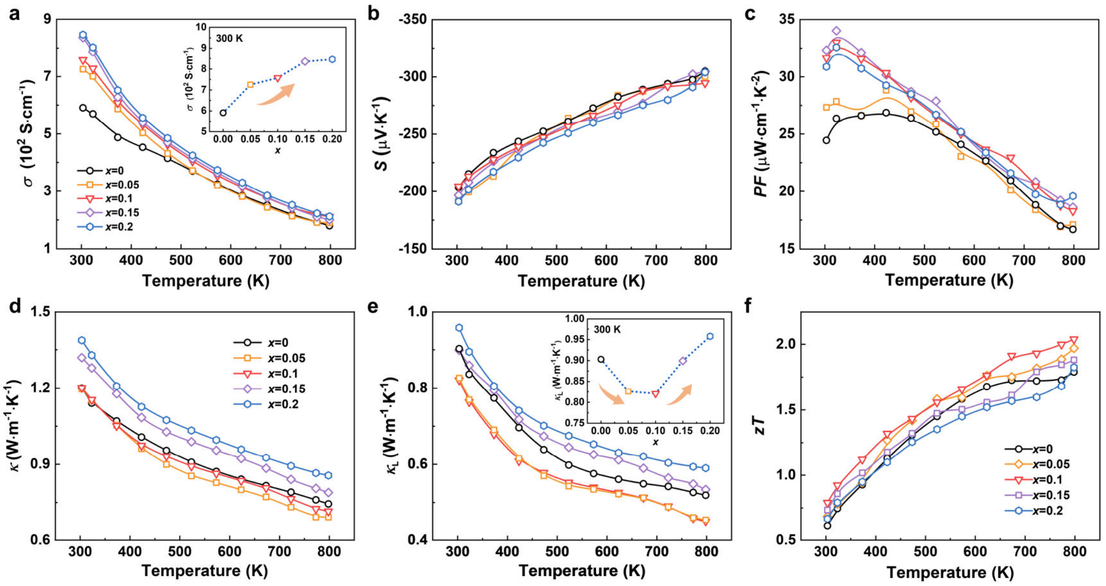

Supplementary Figure 2. Thermoelectric properties of Nb-added samples. Temperature dependence of the (a) electrical conductivity, (b) Seebeck coefficient, (c) power factor, (d) total thermal conductivity, (e) lattice thermal conductivity and (f) zT of xNb/$Mg_3Sb_{1.5}Bi_{0.49}Te_{0.01}$ samples. The insets in (a) and (e) show the electrical conductivity and the lattice thermal conductivity depending on x at 300 K, respectively.

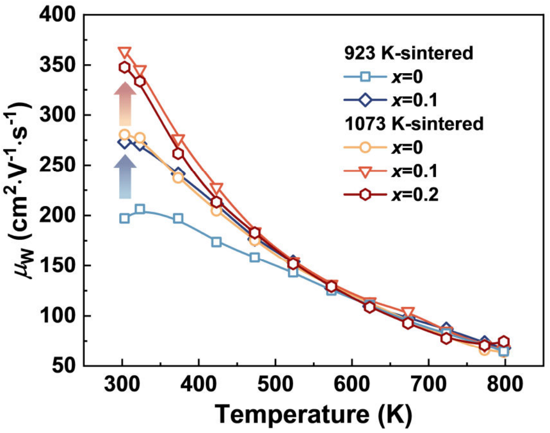

Supplementary Figure 3. Weighted mobility. Temperature dependence of weighted mobility for xNb/$Mg_3Sb_{1.5}Bi_{0.49}Te_{0.01}$ (x = 0, 0.1 and 0.2).

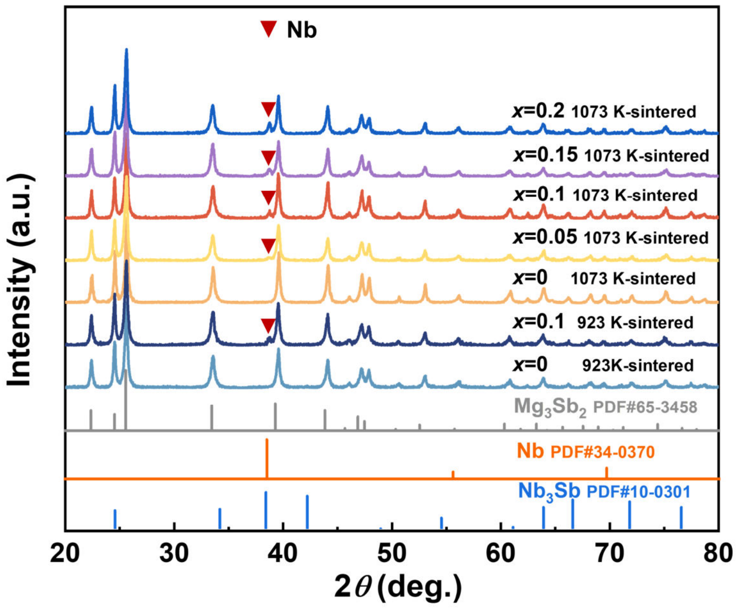

Supplementary Figure 4. Phase characterization. XRD patterns of xNb/$Mg_3Sb_{1.5}Bi_{0.49}Te_{0.01}$ (x = 0 and 0.01) sintered at 923 K and 1073 K.

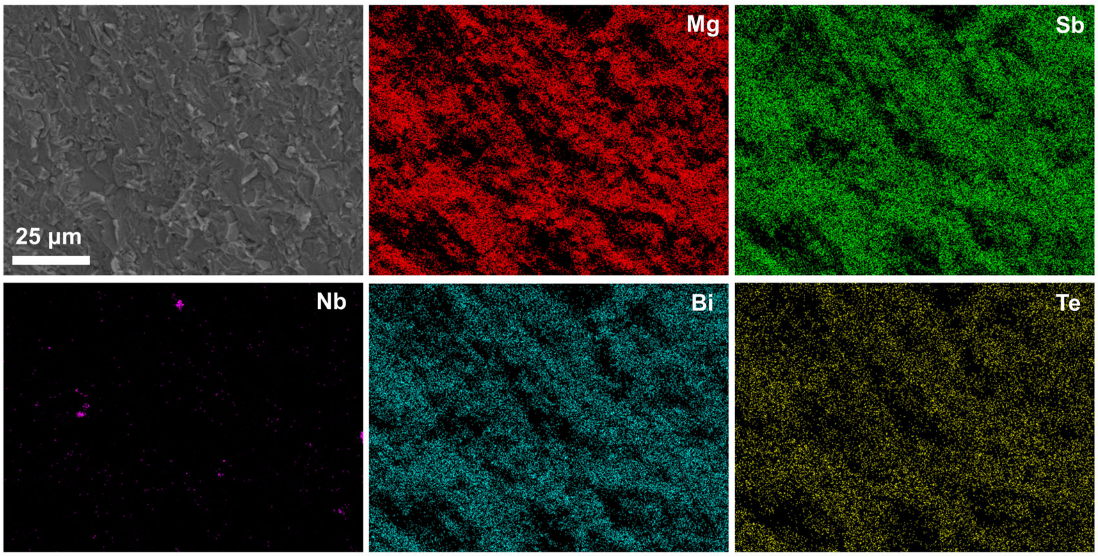

Supplementary Figure 5. The distribution of Nb inclusions. EDS mapping of 0.1Nb/$Mg_3Sb_{1.5}Bi_{0.49}Te_{0.01}$ sintered at 1073 K on the fracture surface.

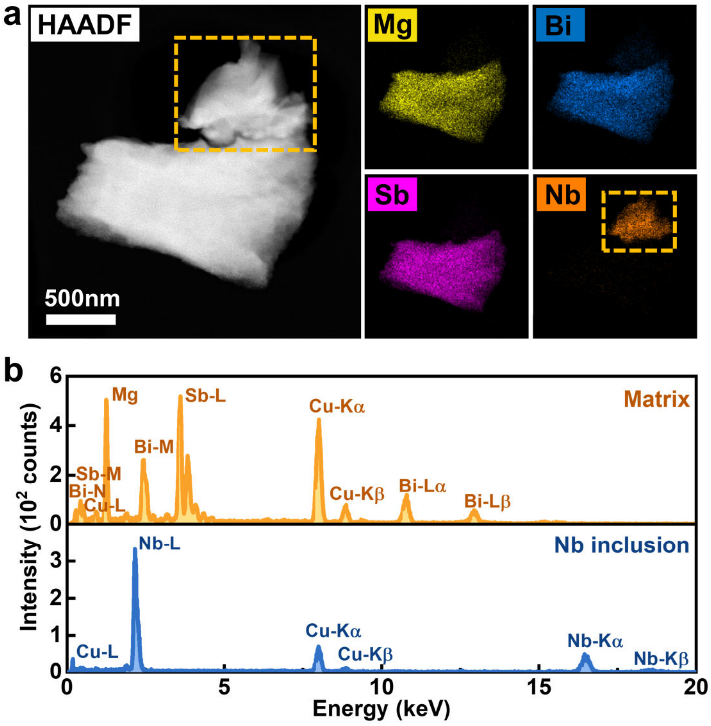

Supplementary Figure 6. Microstructure characterization of the matrix and Nb inclusions. (a) HAADF-STEM mapping of 0.1Nb/$Mg_3Sb_{1.5}Bi_{0.49}Te_{0.01}$ powder sintered at 1073 K. (b) EDS analyses for a matrix grain and Nb secondary phase inclusions from a random area in (a). The same analysis was carried out for about 10 grains, and no Nb signals were detected among them.

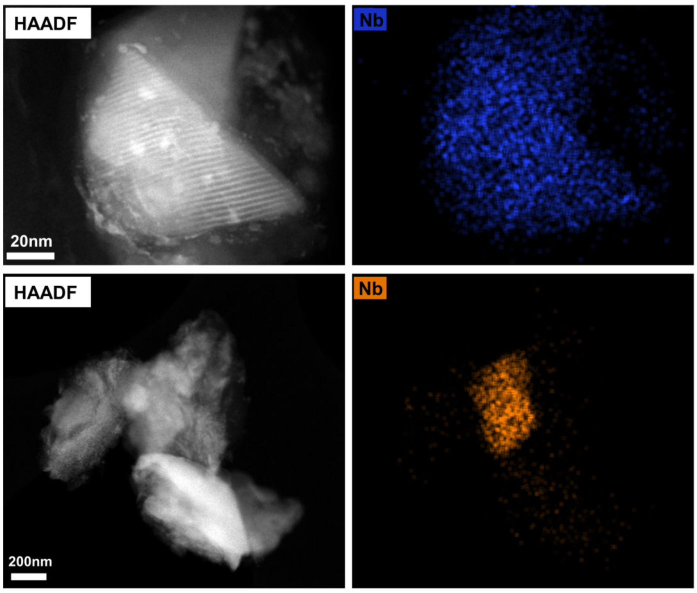

Supplementary Figure 7. The size of Nb inclusions. HAADF-STEM and Nb elemental map images of Nb secondary phase for $0.1Nb/Mg_3Sb_{1.5}Bi_{0.49}Te_{0.01}$.

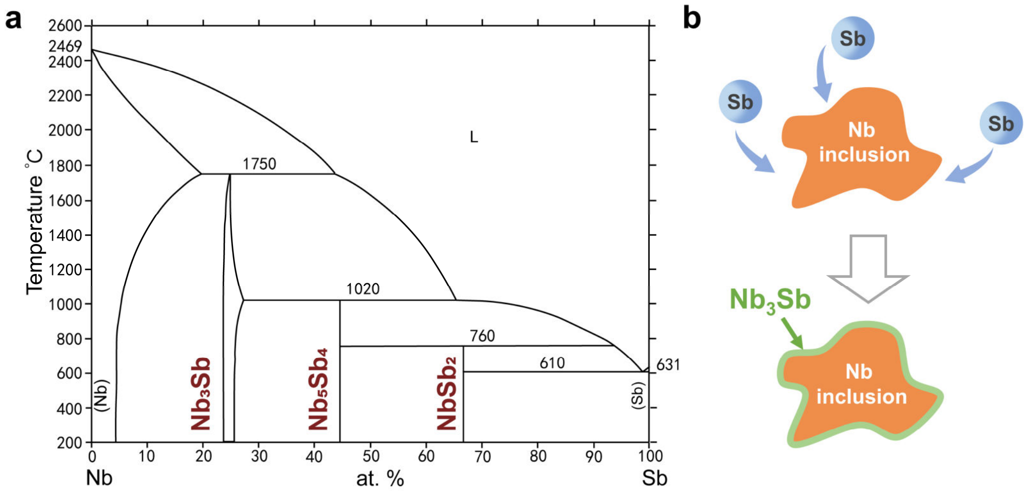

Supplementary Figure 8. The formation mechanism of the secondary phase. a) The Nb-Sb binary phase diagram[30]. b) Schematic illustration of the structure of Nb inclusion.

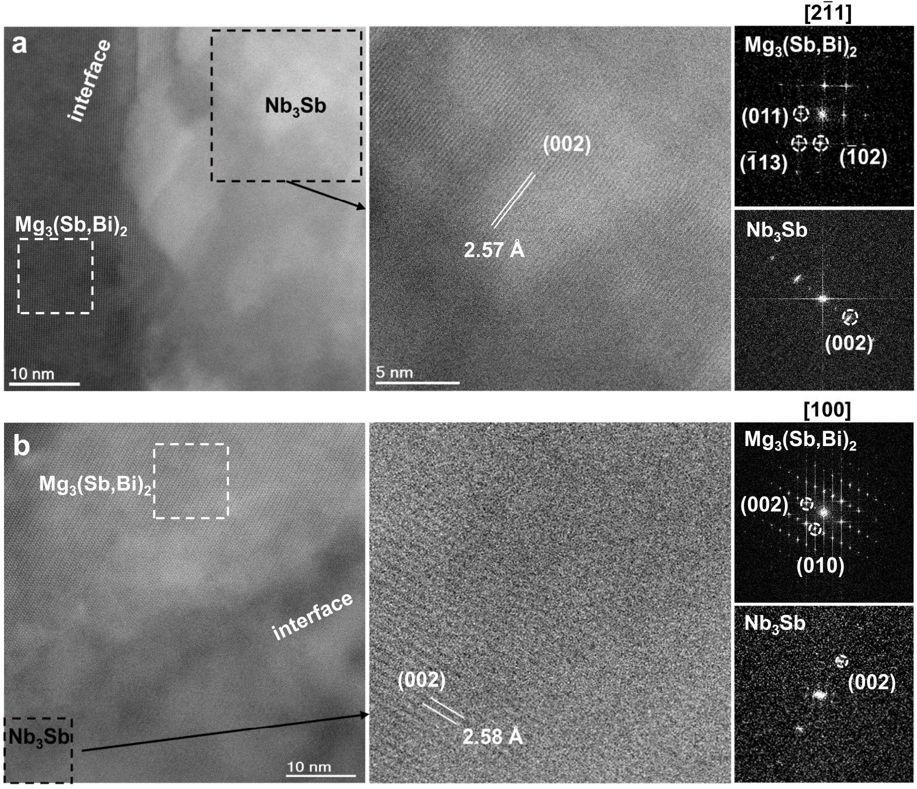

Supplementary Figure 9. Interface characterization. HRTEM images of the interface between the matrix and Nb-rich secondary phase with the FFT images of the boxed region.

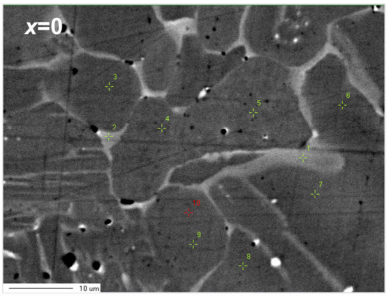

Point	Mg(Atom%)	Sb(Atom%)	Bi(Atom%)	Te(Atom%)	Total(Atom%)
1 (grain boundary)	54.3259	31.4616	14.2125	0.0000	100.0000
2 (grain boundary)	53.5691	31.9092	14.5217	0.0000	100.0000
3 (grain)	54.2892	35.6365	10.0743	0.0000	100.0000
4 (grain)	54.3319	35.4817	10.1864	0.0000	100.0000
5 (grain)	54.6288	34.4767	10.8946	0.0000	100.0001
6 (grain)	54.1782	35.6277	10.1941	0.0000	100.0000
7 (grain)	54.4387	34.4567	11.1046	0.0000	100.0000
8 (grain)	53.9813	35.3714	10.6473	0.0000	100.0000
9 (grain)	54.2115	35.2970	10.4915	0.0000	100.0000
10 (grain)	53.9256	35.8095	10.2649	0.0000	100.0000

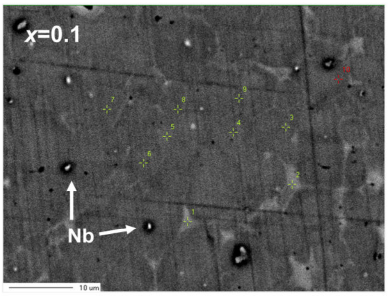

Point	Mg(Atom%)	Sb(Atom%)	Bi(Atom%)	Te(Atom%)	Nb(Atom%)	Total(Atom%)
1 (grain boundary)	55.7656	31.9961	12.1904	0.0000	0.0479	100.0000
2 (grain boundary)	55.8949	31.8074	12.2977	0.0000	0.0000	100.0000
3 (grain)	55.8005	33.5658	10.6337	0.0000	0.0000	100.0000
4 (grain)	56.2770	33.0120	10.7109	0.0000	0.0000	99.9999
5 (grain)	56.1905	33.0293	10.7802	0.0000	0.0000	100.0000
6 (grain)	56.2073	33.3687	10.4240	0.0000	0.0000	100.0000
7 (grain)	56.3386	33.3052	10.3546	0.0000	0.0016	100.0000
8 (grain)	56.0079	33.5004	10.4917	0.0000	0.0000	100.0000
9 (grain)	56.0125	33.6022	10.3671	0.0000	0.0182	100.0000
10 (grain)	55.3863	33.5048	11.0425	0.0000	0.0664	100.0000

Supplementary Figure 10. Composition of the matrix. Back-scattering images from polished surface of xNb/$Mg_3Sb_{1.5}Bi_{0.49}Te_{0.01}$ (x = 0 and 0.01) sintered at 1073 K and corresponding point composition estimated by EPMA. The presence of Nb signals may be due to the residue of Nb inclusions during polishing or Nb inclusions whose size is less than the probe limit of 1μm.

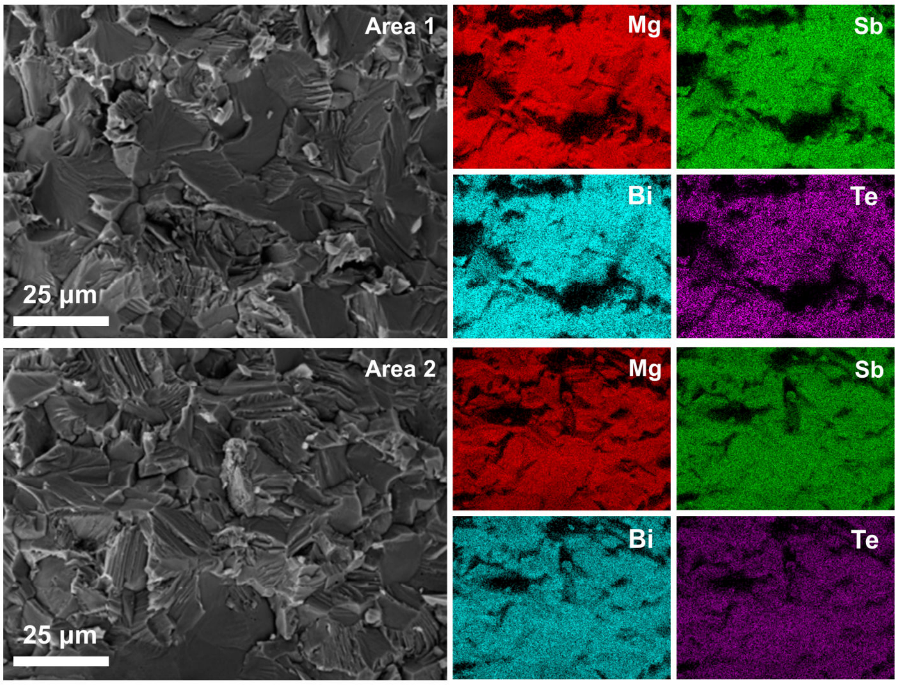

Supplementary Figure 11. **Characterization of the unadded sample.** EDS mapping of different areas on the fracture surface for the unadded sample sintered at 1073 K.

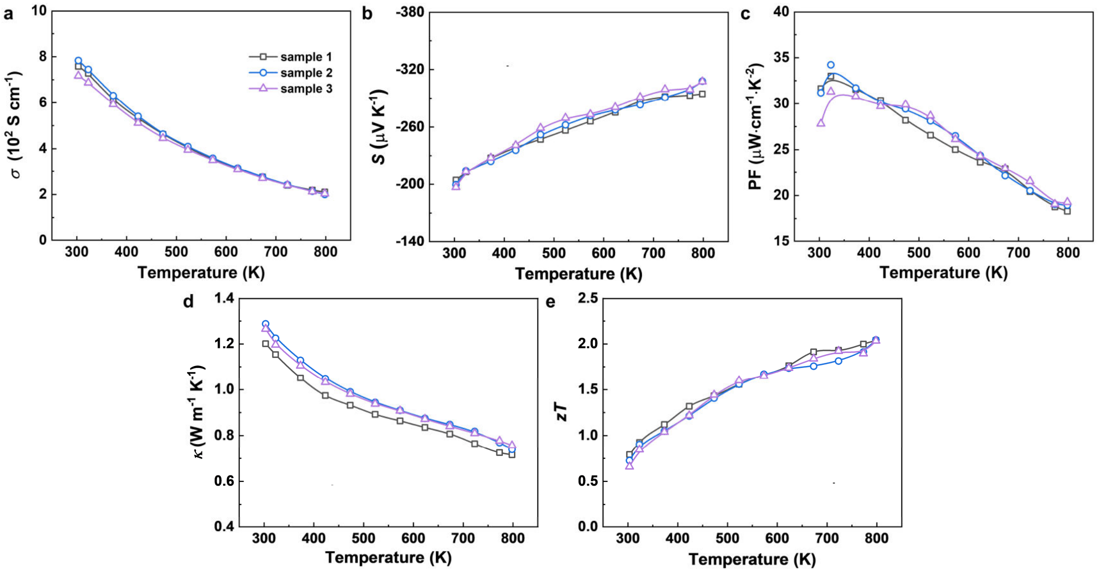

Supplementary Figure 12. Reproducibility of thermoelectric properties for 0.1Nb/$Mg_3Sb_{1.5}Bi_{0.49}Te_{0.01}$. Temperature dependence of (a) electrical conductivity, (b) Seebeck coefficient, (c) PF, (d) total thermal conductivity, and (e) zT.

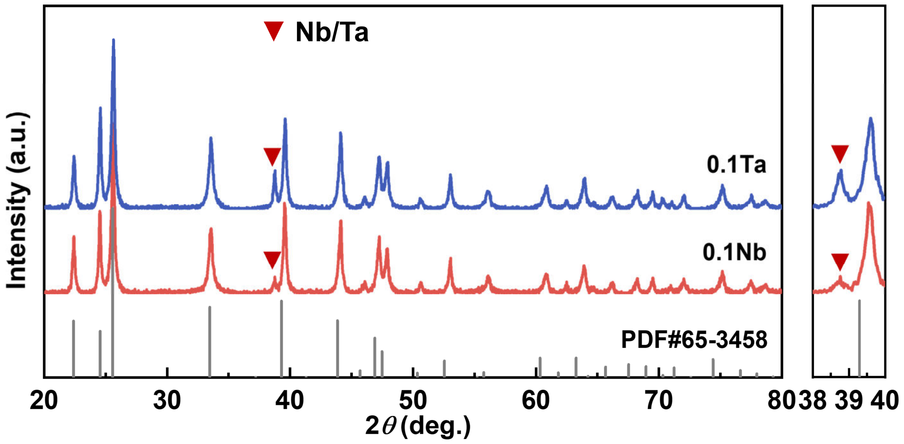

Supplementary Figure 13. Phase characterization of the Nb-added and Ta-added samples. XRD patterns of 0.1Nb/$Mg_3Sb_{1.5}Bi_{0.49}Te_{0.01}$ and 0.1Ta/$Mg_3Sb_{1.5}Bi_{0.49}Te_{0.01}$ sintered at 1073 K, and the enlarged peak between 38° and 40°.

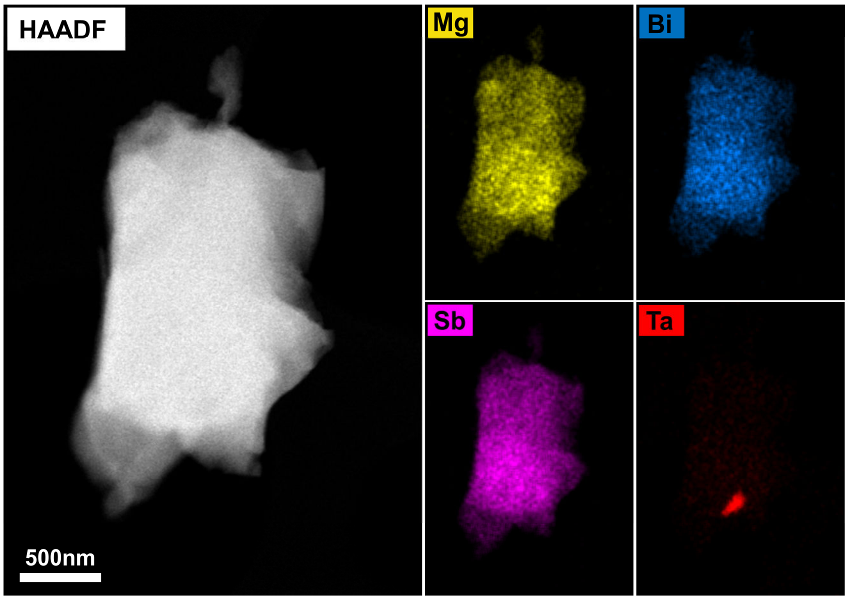

Supplementary Figure 14. Microstructure characterization of Ta inclusions. HAADF-STEM mapping of 0.1Ta/$Mg_3Sb_{1.5}Bi_{0.49}Te_{0.01}$.

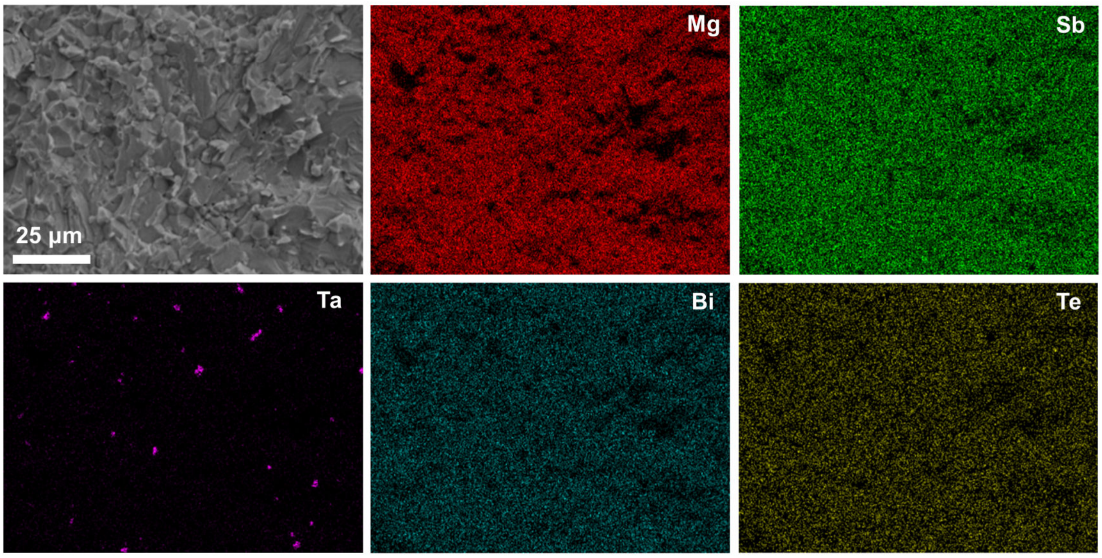

Supplementary Figure 15. The distribution of Nb inclusions. EDS mapping of 0.1Ta/$Mg_3Sb_{1.5}Bi_{0.49}Te_{0.01}$ sintered at 1073 K on the fracture surface.

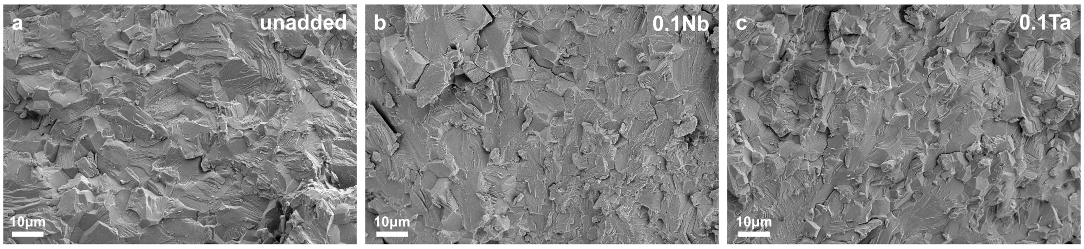

Supplementary Figure 16. Grain Size of the Nb-added and Ta-added samples. SEM images of (a) the unadded sample, (b) the Nb-added sample and (c) the Ta-added sample on the fracture surface.

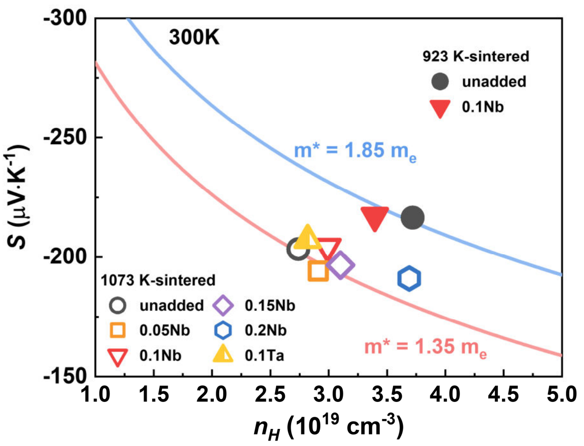

Supplementary Figure 17. Pisarenko plots. The Seebeck coefficients as a function of carrier concentration at 300 K.

For a fixed density-of-state effective mass (m^*) of 1.35 m_e, the data points of Nb-added samples sintered at 1073 K are very close to the curve, but deviate upward slightly with increasing the amount of Nb. The point corresponding to the Ta-added sample also shows a similar deviation. However, both the samples sintered at 923 K are fitted well with a m^* of ~1.85m_e regardless of the addition of Nb. The m^* reduces from ~1.85 m_e to 1.35 m_e with increasing the sintering temperature, which may be attributed to the massive defects generated in $Mg_3Sb_{1.5}Bi_{0.5}$-based materials at higher sintering temperatures. Since the diffusion at the interface sintered at 923 K is not as sufficient as that sintered at 1073 K, the interface may have a weaker contact, leading to less reduction of the interfacial barriers. As a result, there is no significant deviation shown in the curve.

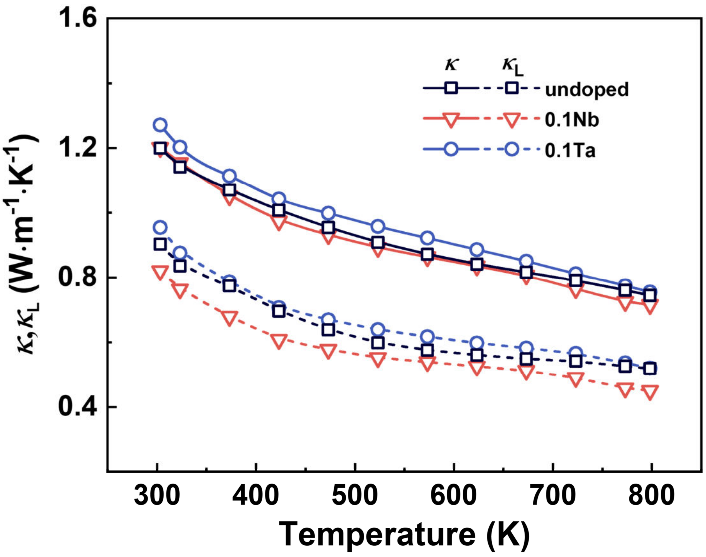

Supplementary Figure 18. Thermal conductivity of the Nb-added and Ta-added samples. Temperature dependence of total and lattice thermal conductivity for $0.1Nb/Mg_3Sb_{1.5}Bi_{0.49}Te_{0.01}$ and $0.1Ta/Mg_3Sb_{1.5}Bi_{0.49}Te_{0.01}$.

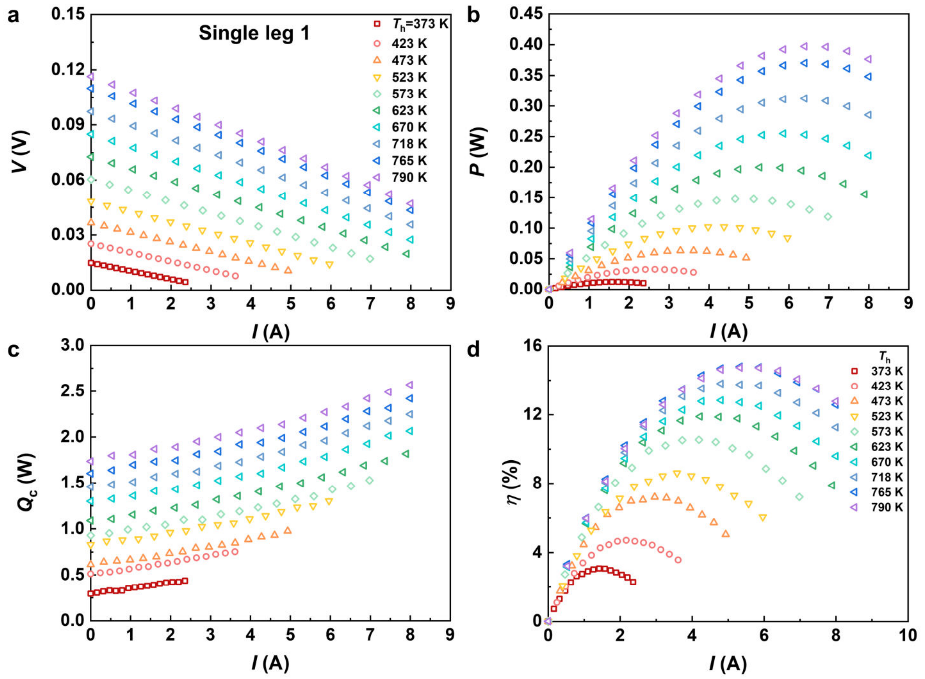

Supplementary Figure 19. Characterization of the single leg 1. The measured (a) voltage, (b) output power (c) output heat and (d) efficiency as a function of the current under different hot-side temperatures for the single leg 1 of 0.1Nb/$Mg_3Sb_{1.5}Bi_{0.49}Te_{0.01}$. The cold-side temperature is 295 K.

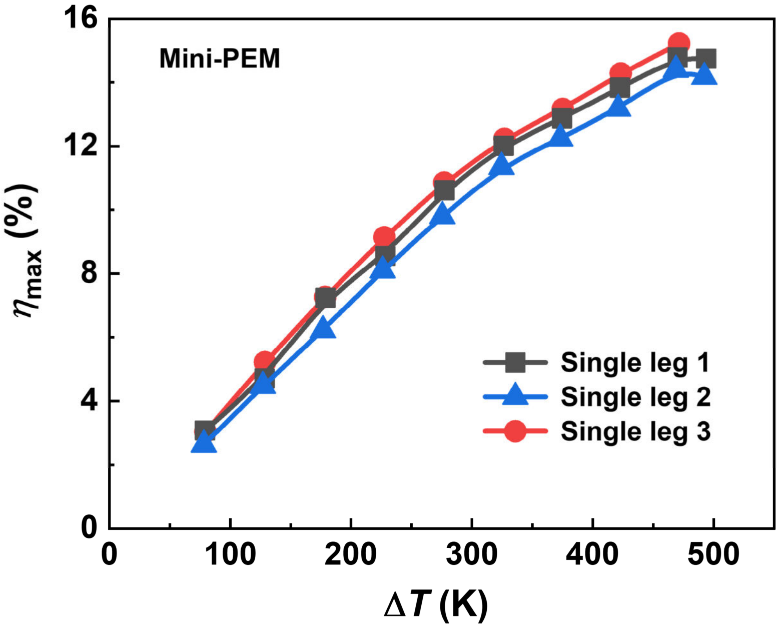

Supplementary Figure 20. Reproducibility of the single legs. Reproducibility of the measured efficiency at different temperatures for the single legs based on the *zT* value of 0.1Nb/$Mg_3Sb_{1.5}Bi_{0.49}Te_{0.01}$.

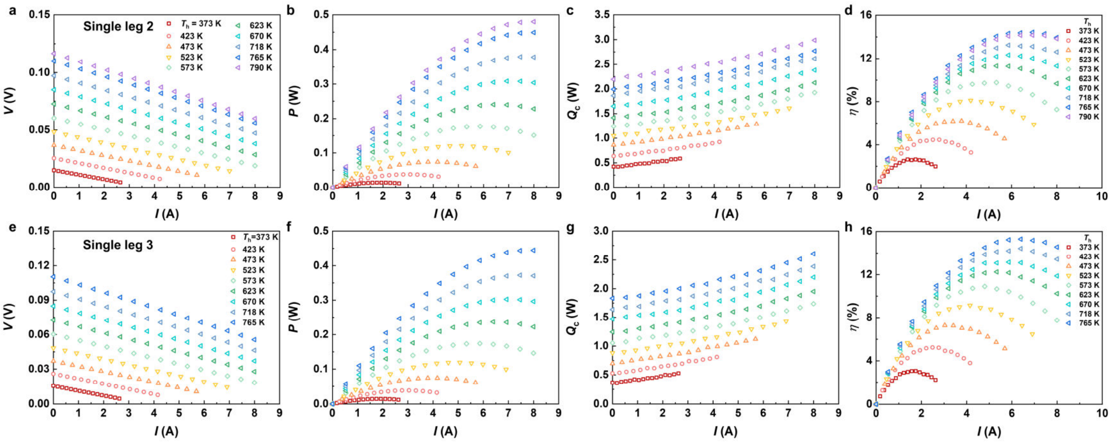

Supplementary Figure 21. Characterization of the single leg 2 and leg 3. The measured (a) voltage, (b) output power (c) output heat and (d) efficiency as a function of the current under different hot-side temperatures for the single leg 2. The cold-side temperature is 295 K. e-h) The measured properties for the single leg 3 and the conditions are the same as in leg 1 and leg 2.

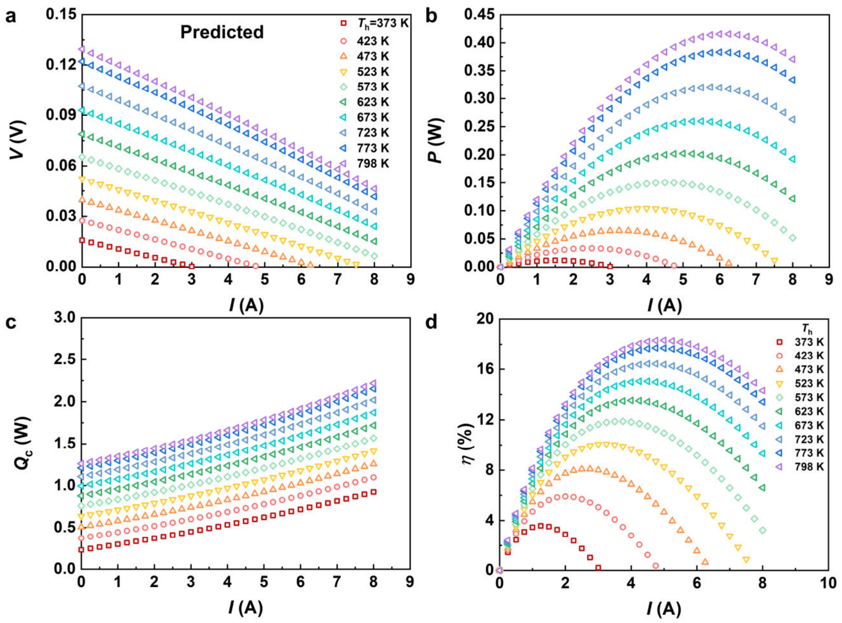

Supplementary Figure 22. Simulation of the single leg. The predicted (a) voltage, (b) output power (c) output heat and (d) efficiency as a function of the current under different hot-side temperatures for the single leg based on the *zT* value of $0.1Nb/Mg_3Sb_{1.5}Bi_{0.49}Te_{0.01}$. The cold-side temperature is 300 K.

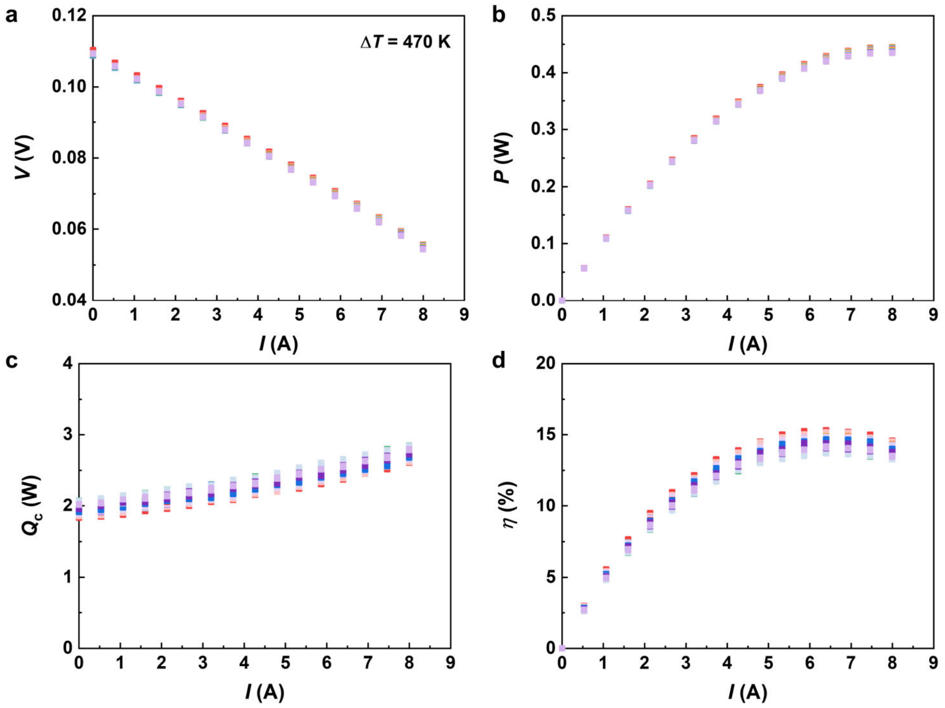

Supplementary Figure 23. Stability of the single leg. Stability of the measured (a) voltage, (b) output power (c) output heat and (d) efficiency as a function of the current under a temperature difference of 470 K and measurement time of 120 h for the single leg of 0.1Nb/$Mg_3Sb_{1.5}Bi_{0.49}Te_{0.01}$. The cold-side temperature is 295 K.

Supplementary Table 1. Room-temperature charge-transport properties for the unadded sample and 0.1Ta/$Mg_3Sb_{1.5}Bi_{0.49}Te_{0.01}$.

**Sample**	σ **(10^2 S cm^{-1})**	S **(μV K^{-1})**	n_H **(10^{19} cm^{-1})**	μ_H **(cm^2 V^{-1} S^{-1})**
unadded sample	5.9	-203	2.74	134.8
0.1Ta	6.3	-207	2.82	140.2
